# Three-Dimensional Au/Holey-Graphene as Efficient Electrochemical Interface for Simultaneous Determination of Ascorbic Acid, Dopamine and Uric Acid

**DOI:** 10.3390/mi10020084

**Published:** 2019-01-24

**Authors:** Aihua Jing, Gaofeng Liang, Yixin Yuan, Wenpo Feng

**Affiliations:** 1 School of Medical Technology and Engineering, Henan University of Science and Technology, Luoyang 471003, China; aihuaj@haust.edu.cn (A.J.); fwp238@haust.edu.cn (W.F.); 2 Medical College, Henan University of Science and Technology, Luoyang 471003, China; 3 Food Science College, Northeast Agricultural University, Harbin 150030, China; yuanyx@haust.edu.cn

**Keywords:** graphene, gold, electrochemical, ascorbic acid, dopamine, uric acid

## Abstract

The quantification of ascorbic acid (AA), dopamine (DA), and uric acid (UA) has been an important area of research, as these molecules’ determination directly corresponds to the diagnosis and control of diseases of nerve and brain physiology. In our research, graphene oxide (GO) with nano pores deposited with gold nanoparticles were self-assembled to form three-dimensional (3D) Au/holey-graphene oxide (Au/HGO) composite structures. The as-prepared 3DAu/HGO composite structures were characterized for their structures by X-ray diffraction, Raman spectrum, scanning electron microscopy, and transmission electron microscopy coupled with cyclic voltammograms. Finally, the proposed 3DAu/HGO displayed high sensitivity, excellent electron transport properties, and selectivity for the simultaneous electrochemical determination of AA, DA and UA with linear response ranges of 1.0–500 μM, 0.01–50 μM and 0.05–50 μM respectively. This finding paves the way for graphene applications as a biosensor for detecting three analytes in human serum.

## 1. Introduction

Ascorbic acid (AA), dopamine (DA), and uric acid (UA) are important molecules that exist in human body fluids, and play an important role in the diagnosis and control of nerve and brain diseases [1]. Their abnormal levels are usually a symptom of illnesses such as schizophrenia, Parkinson’s, gout, scurvy, and cardiovascular diseases [2,3]. Moreover, their oxidation potentials as common solid electrodes are close to each other, and it is therefore necessary to detect them simultaneously in order to understand the mechanism for biomedical and diagnostic pathology. A wide variety of analytical techniques, including fluorescence [4,5], chemiluminescence [6], and electrochemistry [7] have been employed for the simultaneous determination of these biomolecules. Among them, electrochemical biosensors based on a modified electrode with an interfacial structures are widely considered the most effective solution to obtain well-separated peaks for their determination because AA, DA, and UA are electroactive and easily oxidizable [8]. Many interfacial structures with large areas and high electronic conductivities—especially carbon-based materials such as mesoporous nitrogen/carbon [9], polymeric/multi-walled carbon nanotubes [1], graphene oxide/zinc sulfide [9,10], graphene fibers [11], and carbon black [12]—have been investigated for their efficacy in the simultaneous electrochemical determination of these biomolecules due to their remarkable electrocatalytic properties. Among them, graphene composites such as vertically aligned ZnO nanowire arrays on three-dimensional (3D) graphene foam [13], 3D graphene hydrogel–gold nanocomposite [14], graphene oxide-templated polyaniline microsheets [15], 3D hierarchical bayberry-like Ni@carbon hollow nanosphere/rGO [16], porphyrin-modified graphene [17], graphene and MgO nanobelts deposited tantalum wire [18], and ferrocene hybrid/3D graphene hydrogel [19] display outstanding sensitivity and selectivity. New electrochemical sensors fabricated from graphene composites with excellent conductivity and large areas still need to be developed for diagnostic and analytical applications. 

Three-dimensional graphene interfacial materials have been widely used as supporting materials in electrochemical capacitors [20], fuel cells [21], solar cells [22], and energy-storage devices [23] because they can enable fast electron transfer and rapid mass transport with large surface areas. The introduction of pores into graphene can tune permeability by allowing certain molecules to pass through [24]. Anchoring nanoscale noble metal particles onto specific supports is a wise strategy for keeping their highly active centers from avoiding aggregation [25]. Three-dimensional graphene interfacial structures serving as electrode materials are expected to show improved electron transfer and mass transport during the electrochemical detection of small biomolecules. They have been prepared by the fabrication of graphene or by chemical vapor deposition (CVD) approaches [26]. However, the low-yield production of CVD-prepared 3D carbon interfacial structures and/or quite expensive and/or unfriendly fabrication processes still hinders their wide application [27]. There is therefore great interest and importance in developing a new approach to synthesizing 3D carbon structures. 

**Scheme 1:**
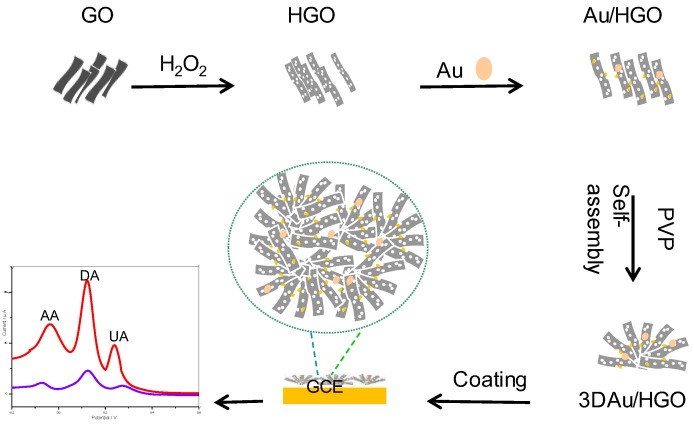
Schematic representation for the preparation of three-dimensional (3D) Au/holey-graphene oxide (Au/HGO)/glass carbon electrode (GCE) biosensor for simultaneous detection of ascorbic acid (AA), dopamine (DA), and uric acid (UA). PVP: polyvinyl pyrrolidone.

Here, three-dimensional composite structures of gold nanoparticles with deposited graphene oxide with nano pores were self-assembled with high conductivity and large active areas, and used for the simultaneous electrochemical determination of AA, DA, and UA. A graphic illustration of the fabrication and detection strategy is displayed in Scheme 1. Low detection limit, high sensitivity, and good repeatability were all observed due to improved electron transfer and mass transport. Additionally, the detection in serum obtained satisfactory recoveries, indicating the potential for further practical application of this sensor. The graphene-based advanced structures open a new avenue for electrochemical biosensors for use in new fields.

## 2. Materials and Methods

### 2.1. Regents and Apparatus

Graphite powder was prepared in our lab and ultra-sonicated to 0.1 mg·mL^−1^. Ascorbic acid (AA) was obtained from Tianjin Fengchuan Reagent Company (Tianjin, China). Dopamine was obtained from Nanjing Oddfoni Biological Technology Company (Nanjing, China). Uric acid was obtained from Sangon Biotech (Shanghai, China). All other reagents were of analytical grade and used as received without further purification unless otherwise specified. Phosphate-buffer solutions (PBS) were prepared by mixing solutions of NaH_2_PO_4_ and Na_2_HPO_4_ and then adjusted with 100 mM NaOH or H_3_PO_4_. Human serum was from Luoyang Blood Center. All the solutions were prepared with twice-distilled water.

The morphologies were characterized by field emission scanning electron microscope (SEM, JEOL JSM-7800F, JEOL Ltd, Tokyo, Japan) and transmission electron microscope (TEM, JEOL JTM-2100, JEOL Ltd, Tokyo, Japan). X-ray diffraction (XRD) measurements were conducted by D8 ADVANCE X-ray diffractometer (Bruker AXS Ltd., karlsruhe, Germany). The Raman spectrum of graphene was recorded on a Renishaw Raman microscope with an excitation laser wavelength at 532 nm. All electrochemical measurements were performed on a CHI660E workstation (Chenhua Instruments, Shanghai, China) with a three-electrode system. Three-dimensional Au/holey-graphene oxide (3DAu/HGO)/glass carbon electrode (GCE) or Au/HGO/GCE was used as the working electrode. Solutions were degassed with nitrogen to remove O_2_. Differential pulse voltammograms (DPVs) were recorded at 0.1 M PBS (pH 7.0) at different concentrations of AA, DA, and UA. For the detection of real samples, a human serum sample was diluted with 0.1 M PBS (pH 7.0) and then transferred to the electrochemical cell for electrochemical determination.

### 2.2. Preparation of Holey Graphene Oxide (HGO)

HGO was synthesized according to previously reported procedure [28]. Typically, 5 mL of 30% H_2_O_2_ aqueous solution was mixed with 50 mL 2 mg·mL^−1^ GO aqueous dispersion and then heated at 100 °C for 2 h under stirring. The as-prepared HGO was purified by centrifuging and washing with pure water to remove the residual H_2_O_2_, and then re-dispersed in pure water for 10 mins to be a dark solution of HGO dispersion (2 mg·mL^−1^).

### 2.3. Preparation of Polyvinyl Pyrrolidone (PVP)-Protected Au Colloids

Typically, 10 mL of 2.0 mM HAuCl_4_ solution, 30 mL of H_2_O, and 0.0889 g of polyvinyl pyrrolidone (PVP, average molecular weight 40,000) was mixed homogeneously. Then, 2.4 mL of 0.1 M NaBH_4_ aqueous solution was quickly added to the above solution. The mixture was stirred for 5 h, and the resulting solution of Au colloids protected by PVP was obtained [29]. 

### 2.4. Synthesis of Au/Holey Graphene Oxide (Au/HGO)

The precursors of Au were dissolved in deionized water to produce a corresponding solution with a requisite concentration of 10 mM directly. First, 30 mg of HGO was dispersed in 60 mL of water and ultra-sonicated to form a homogeneous aqueous solution. Added into the above HGO suspension was 1.69 mL 10 mM Au precursor solution. The whole process was performed in an ice bath and kept stirring for 30 min. After centrifugation at 12,000 rpm for 30 min and washing with deionized water 3–4 times until the ion concentration of the supernatant was less than 10 ppm, Au/HGO was obtained [25].

### 2.5. Preparation of PVP/Au/HGO

In a typical procedure, 80 mg of PVP was added into 100 mL of Au/HGO dispersion (0.25 mg·mL^−1^), followed by stirring for 12 h. Thirty-five microliters of hydrazine solution (50% *w*/*w*) and 400 μL of ammonia solution (25% *w*/*w*) were then added to the resulting dispersion. After being vigorously shaken or stirred for a few minutes, the mixture was stirred for 1 h at 95 °C. Finally, the stable black dispersion was centrifuged two times and dissolved in 25 mL of water (1 mg·mL^−1^) [30].

### 2.6. Preparation of 3D Au/HGO Modified Glassy Carbon Electrode (3D Au/HGO/GCE)

A glass carbon electrode (GCE) was polished until mirror-like with 0.3 and 0.05 μm alumina slurry (Beuhler) followed by sonicating in acetone, nitric acid solution (1:1, *v*/*v*), and pure water. The electrode was dried at 4 °C for 24 h after 20 μL of 1.0 mg·mL^−1^ PVP/Au/HGO was dropped on the surface for self-assembly to 3DAu/HGO/GCE for further electrochemical investigation.

## 3. Results

### 3.1. Characterization of 3DAu/HGO

**Figure 1:**
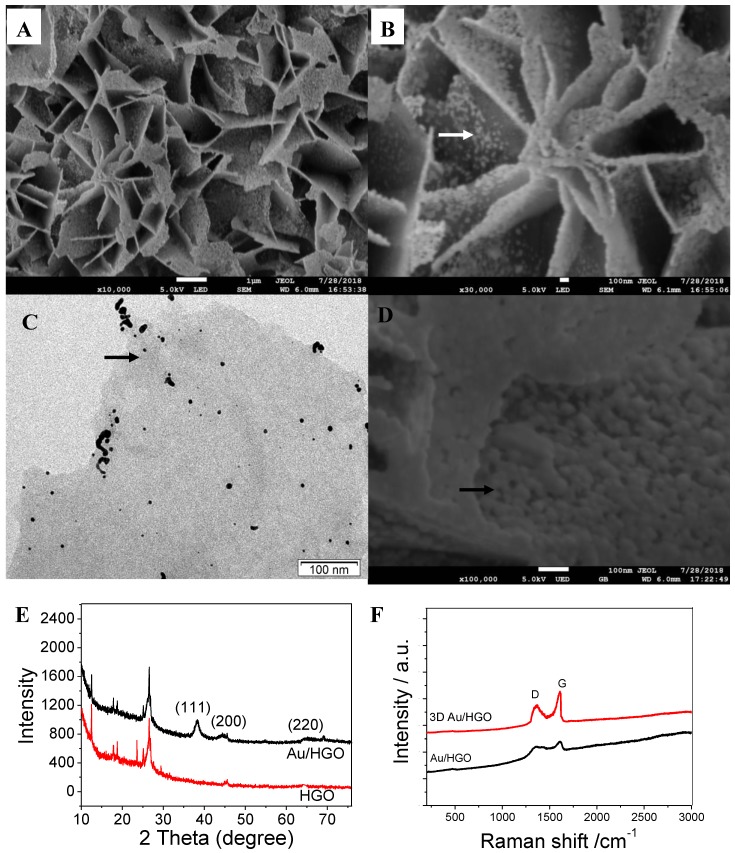
(**A**) Low- and (**B**) high-magnification SEM images of as-prepared 3DAu/HGO. (**C**) TEM and (**D**) SEM of Au/HGO. (**E**) X-ray diffraction (XRD) patterns and (**F**) Raman spectrum of 3DAu/HGO and holey-graphene oxide (HGO). Arrows indicate gold nanoparticles.

Figure 1A,B shows the morphology of the as-prepared 3DAu/HGO, which was found to have an obvious three-dimensional porous structure, with plates piling up to a flower-like shape with a larger specific surface area (Figure S1 shows the role of PVP). The TEM of Figure 1C and the SEM of Figure 1D display gold nanoparticles homogeneously decorated on the graphene sheets, with a size of tens of nanometers. The influence of the concentration of Au precursor on the structures of Au/HGO is shown in Figure S2. After increasing the concentration of Au precursor, HGO was decorated by more and larger gold nanoparticles. 

The structural characteristics of the as-prepared 3DAu/HGO composite structures were investigated by X-ray diffraction (XRD), as shown in Figure 1E. The diffraction peaks observed at 25.2° were ascribed to HGO. The diffraction peaks at 38.2°, 44.3°, and 64.5° corresponded to (111), (200), and (220) crystal faces of the Au nanoparticles respectively. The characteristic peaks for Au nanoparticles of 3DAu/HGO composite structures were much weaker, and could be ascribed to the weight ratio of gold in the as-synthesized composite structures. They may have been relatively low, or gold nanoparticles in the as-prepared Au/HGO composite structures could have been homogeneously well-dispersed on the HGO surface [25].

Figure 1F shows the Raman spectra of 3DAu/HGO and HGO. The spectral features include a D band at approximately 1349 cm^−1^ and G band at around 1576 cm^−1^. The D band is a characteristic feature, indicating defects. It can be caused by excitation of the edges of graphene sheets and their random orientation, which corresponds to the vibration of sp^3^ and sp^2^-hybridized carbon atoms. Because the intensity of the G peak was somewhat stronger than that of the D peak, the 3DAu/HGO had a much higher intensity ratio of D band to G band (D/G) in comparison with Au/HGO, which is attributed to the increase in the spatially ordered crystal structure of graphene. This interconnected porous structure enables fast electron transfer in electrochemical applications [26,31].

### 3.2. 3DAu/HGO for the Electrochemical Determination of AA, DA, and UA

**Figure 2:**
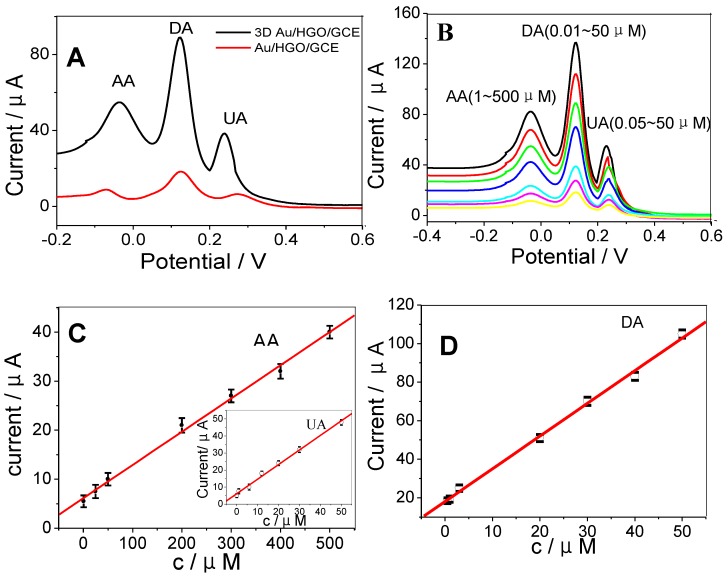
(**A**) Differential pulse voltammograms (DPVs) at Au/HGO/GCE and 3D Au/HGO/GCE containing 400 μM AA, 10 μM DA, and 15 μM UA at a scan rate of 50 mV·s^−1^. (**B**) DPVs of 3D Au/HGO/GCE for the simultaneous determination of AA, DA, and UA in 0.1 M phosphate-buffered saline (PBS). Calibration curves of 3DAu/HGO/GCE for simultaneous determination of (**C**) AA, (**D**) DA, and UA (inset of (C)). The points on calibration curves correspond to different concentrations of AA, DA, and UA which are displayed in different colors in Figure 2B.

**Scheme 2:**
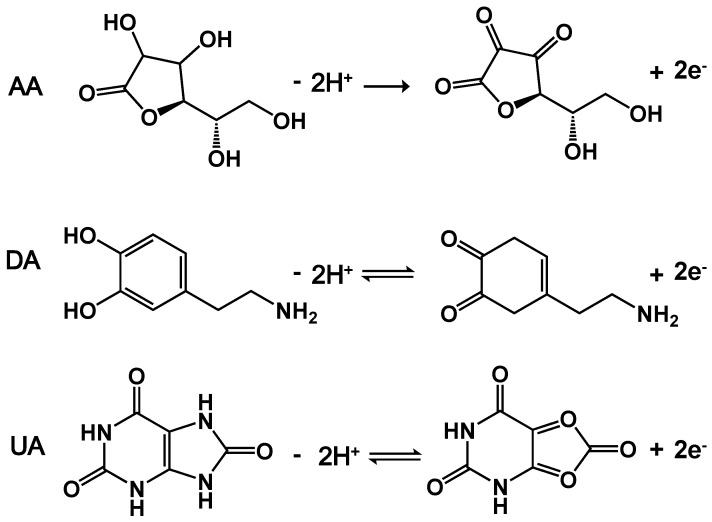
Electrochemical reaction mechanism for AA, DA, and UA on 3DAu/HGO electrode.

Figure 2A shows DPVs of Au/HGO/GCE and 3DAu/HGO/GCE in 0.1M PBS containing 400 μM AA, 10 μM DA, and 15 μM UA. For the 3DAu/HGO/GCE, three well-separated anodic peaks were observed at −0.276, 0.156, and 0.340 V, corresponding to the electro-oxidation of AA, DA, and UA, respectively. The anodic peak potential differences were 0.432 V between AA and DA, 0.184V between DA and UA, and 0.616 V between AA and UA. Moreover, the oxidation current densities for these three biomolecules at 3DAu/HGO/GCE were much higher than those at Au/HGO/GCE. The experiments confirmed that 3DAu/HGO/GCE possessed good electrocatalytic activity towards the oxidation of these molecules. The enhanced electrocatalytic activity of 3DAu/HGO/GCE could probably be ascribed to the unique highly porous 3D structure of its large electrochemically active surface area, which enabled the rapid and fast electron transport of analytes to the electrode surface (as shown in Figure S1). Scheme 2 displays the mechanism of reactions between AA, DA, UA, and 3DAu/HGO/GCE. It can be seen from Scheme 2 that when the oxidation of hydroxyl groups of the furan ring changed to carbonyl groups in AA, the oxidation of catechol changed to o-quinone in DA, and the oxidation of bridging double-bond changed to hydroxyl groups in UA took place, anodic peak responses could be observed by increases on the electrode. Furthermore, two protons and two electrons were involved in the oxidation processes.

**Figure 3:**
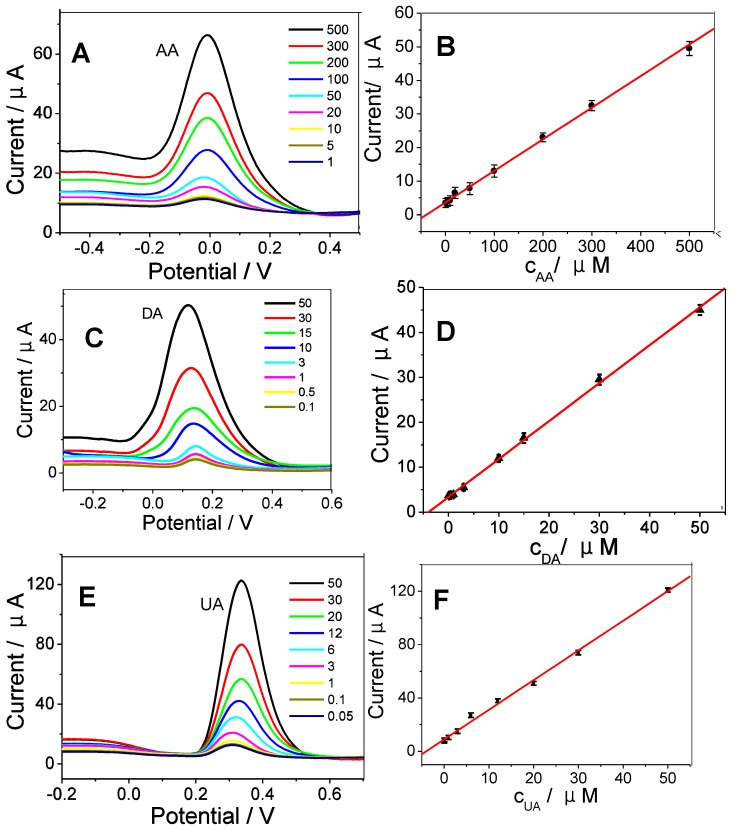
DPVs for 3DAu/HGO/GCE under increased concentrations of (**A**) AA, (**C**) DA, and (**E**) UA. Calibration curves for (**B**) AA, (**D**) DA, and (**F**) UA. Electrochemical parameters were the same as in Figure 2B.

The simultaneous determination of AA, DA, and UA was carried out in PBS buffer solutions (Figure 2B). The calibration curves were linear with the following concentration ranges: 1.0–500 μM for AA, 0.01–50 μM for DA, and 0.05–50 μM for UA. The LODs (limit of detections) for AA, DA, and UA were 0.1, 0.005, 0.01 μM, respectively. Figure 2C,D shows the calibration curves at increased concentrations of AA, DA, and UA obtained on 3DAu/HGO/GCE. The individual detections of AA, DA, and UA are shown in Figure 3A,C,E. The linearity range for AA, DA, and UA obtained in the separated measurements, displayed in Figure 3B,D,F, was the same as that in the simultaneous measurements. The LOD obtained in the separated measurements was comparable with that in the simultaneous measurements, indicating that 3DAu/HGO/GCE had an excellent ability for the simultaneous electrochemical determination of AA, DA, and UA. These results were compared with other nanomaterial-modified electrodes in the literature, and the results are summarized in Table 1. The 3DAu/HGO/GCE exhibited broader linear range and lower detection limit for AA, DA, and UA than most of the electrodes listed below. 

The relative standard deviations for three continuous measurements were 3.2%, 4.0%, and 2.9% for AA, DA, and UA, respectively, in 0.1 M PBS buffer solutions containing 400 μM AA, 10 μM DA, and 15 μM UA. In addition, the 3DAu/HGO/GCE showed no obvious current response decay after being stored in 0.1 M PBS at 0 °C for two weeks, indicating the good stability of the 3DAu/HGO/GCE electrode. 

### 3.3. Real Sample Analysis

To examine the application of the 3DAu/HGO/GCE electrode, it was used for the detection of AA, UA, and DA in human serum samples using the standard addition method. Each sample was diluted five times with 0.1 M PBS (pH 7.0) before measurement. The satisfactory recoveries obtained were between 94.7% and 106% for all the analytes (Table 2). These results confirmed that the 3DAu/HGO composite structures are a good material for the detection of small biomolecules with low detection limits and high sensitivities. The 3DAu/HGO composite structures can meet real application requirements in the linear range after simple dilution. 

## 4. Conclusions

In this study, graphene oxide with nano pores was prepared via a convenient mild defect-etching reaction, and then gold nanoparticles were deposited on it. The resulting gold nanoparticle-deposited holey-graphene oxide was self-assembled on glass carbon electrodes thereafter to form three-dimensional composite structures for sensing. Results showed high specific surface area and high electrical conductivity in the simultaneous electrochemical detection of AA, DA, and UA. Ultra-low detection limits and high sensitivity were obtained. In addition, the electrode successfully detected human serum samples. This work demonstrates an electrode material that is superior for the electrochemical detection of biomolecules in practical analysis. 

## Figures and Tables

**Table 1:** Comparison of different carbon-based materials for the simultaneous determination of AA, DA, and UA.

Modified Electrode	Linear Range (μmol·L^−1^)			Detection Limit (μmol·L^−1^)			Ref.
	AA	DA	UA	AA	DA	UA	
Polymer/Multi-walled Carbon Nanotubes	3.0–1500	10–600	2.0–60	1.65	2.01	0.46	[1]
Mesoporous Nitrogen/Carbon	1.0–700	0.001–30	0.01–80	0.01	0.001	0.01	[9]
Graphene Oxide/Zinc Sulfide	50–1000	1.0–500	1.0–500	30	0.5	0.4	[10]
Flexible Graphene Fibers	200–750	1.0–13	10–260	50	0.1	0.2	[11]
Carbon Black	1.02–20.2	0.9–18.6	0.79–11.7	0.008	0.117	0.138	[12]
3DAu/HGO	1.0–500	0.01–50	0.05–50	0.1	0.005	0.01	This work

**Table 2:** Recovery of AA, DA, and UA in real samples (*n* = 5).

Samples	Added (μM·L^−1^)			Found (μM·L^−1^)			Recoveries (%)		
	AA	DA	UA	AA	DA	UA	AA	DA	UA
Serum 1	10.00	1.00	5.00	10.19	9.81	5.19	101.9	98.1	103.8
Serum 2	30.00	5.00	10.00	31.80	5.12	9.93	106.0	102.4	99.3
Serum 3	150.00	30.00	30.00	142.05	30.35	28.86	94.7	101.2	96.2

## Supplementary Materials

The following are available online at http://www.mdpi.com/2072-666X/10/2/84/s1, Figure S1: 3D Au/HGO self-assembly by Au/HGO protected (**a**) with PVP and (**b**) without PVP; Figure S2: TEM of Au/HGO Prepared with Different Concentrations of Au Precursor: (**a**) 5 mM, (**b**) 10 mM, and (**c**) 20 mM.
