# Supplementary material for: Three-Dimensional Au/Holey-Graphene as Efficient Electrochemical Interface for Simultaneous Determination of Ascorbic Acid, Dopamine and Uric Acid
Source: Micromachines (Basel). 2019 Jan 24;10(2):84. doi: 10.3390/mi10020084 (PMC6413087; doi:10.3390/mi10020084)
Supplement: Supplementary file 1 [file micromachines-10-00084-s001.pdf]

Supplementary Materials: Three-Dimensional Au/Holey-Graphene as Efficient Electrochemical Interface for Simultaneous Determination of Ascorbic Acid, Dopamine and Uric Acid

Aihua Jing, Gaofeng Liang, Yixin Yuan and Wenpo Feng

1. Role of PVP

Polyvinyl pyrrolidone (PVP) is a water-soluble polymer made from the monomer *N*-vinylpyrrolidone. PVP has a structure of a polyvinyl skeleton with polar groups, shown in the formula:

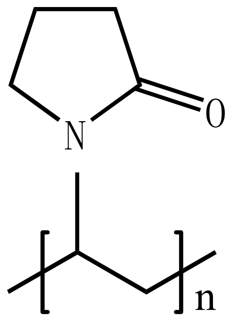

where *n* is the polymerization number.

The advantage of PVP over other water-soluble polymers is that PVP molecules can disperse uniformly on graphene sheets after solvent evaporation, and then turn into three-dimensional structures—a change ascribed to a strong ð–ð interaction between PVP and graphene. By comparison, pure graphene sheets are only connected by a weak physical interaction, which can turn into amorphous structures due to a reduction in the interfacial thermal resistance.

Figure S1 is a three-dimensional (3D) Au/holey-graphene oxide (Au/HGO) self-assembly by Au/HGO protected (a) with PVP and (b) without PVP.

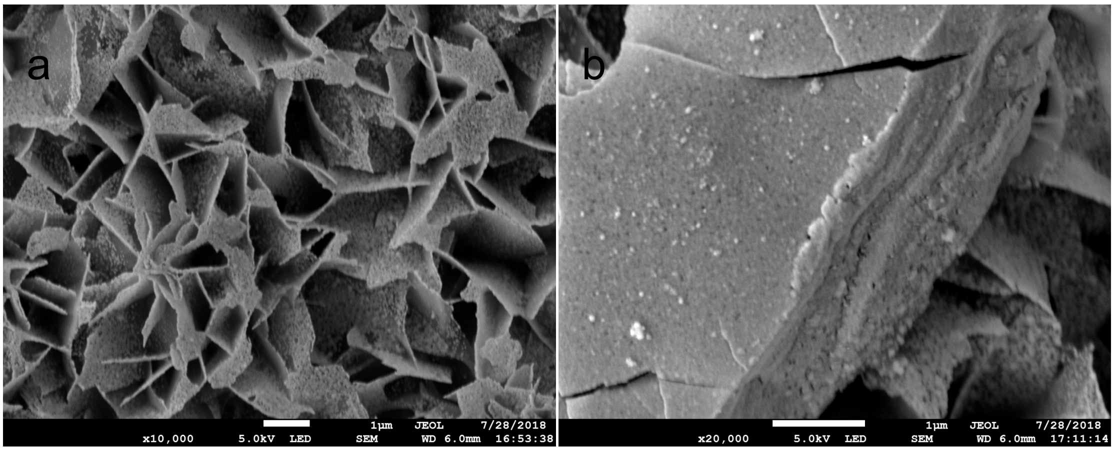

Figure S1. 3D Au/HGO self-assembly by Au/HGO protected (**a**) with PVP and (**b**) without PVP.

2. Preparation of Au/HGO-Modified Glassy Carbon Electrode (Au/HGO/GCE)

A glass carbon electrode (GCE) was polished until mirror-like with 0.3 and 0.05 μm alumina slurry (Beuhler), followed by sonicating in acetone, nitric acid solution (1:1, v/v), and pure water.

Then, 20 μL of 1.0 mg·mL^{-1} Au/HGO was cast-coated on a clean glass carbon electrode (GCE) (diameter: 3 mm) and dried in air.

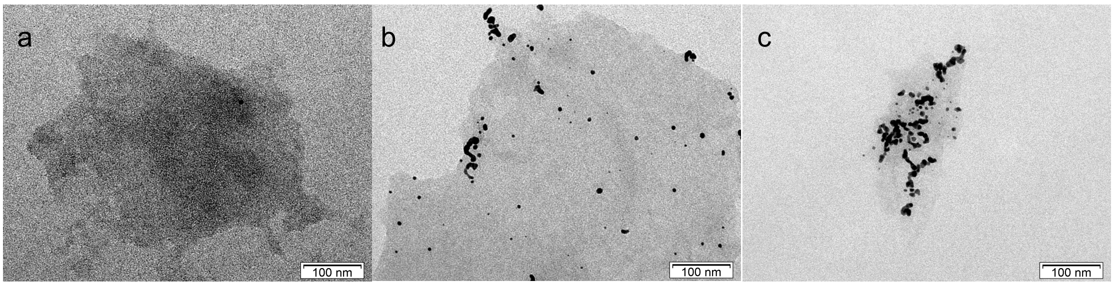

Figure S2. TEM of Au/HGO Prepared with Different Concentrations of Au Precursor: (**a**) 5 mM, (**b**) 10 mM, and (**c**) 20 mM.
